# Supplementary material for: Ecological Requirements for Abundance and Dispersion of Brazilian Yellow Fever Vectors in Tropical Areas
Source: Int J Environ Res Public Health. 2024 May 10;21(5):609. doi: 10.3390/ijerph21050609 (PMC11120827; doi:10.3390/ijerph21050609)
Supplement: Supplementary file 1 [file ijerph-21-00609-s001.zip › ijerph-2950507-supplementary.pdf]

Supplementary material

Table S1. Concurrent models to analyze how particular landscape metrics influence the abundances of *Haemagogus leucocelaenus* and *Haemagogus janthinomys* at different scales (500 m, 1.0 km, 1.5 km, 2.0 km, and 2.5 km).

Species	Predictor variables	Scales
Haemagogus leucocelaenus/ Haemagogus janthinomys	null 1 = abundance ~1	-
Haemagogus leucocelaenus/ Haemagogus janthinomys	null 2 = abundance ~ latitude	-
Haemagogus leucocelaenus/ Haemagogus janthinomys	null 3 = abundance ~ longitude	-
Haemagogus leucocelaenus/ Haemagogus janthinomys	null 2 = abundance ~ latitude + longitude	-
Haemagogus leucocelaenus/ Haemagogus janthinomys	Percentage of forest cover (1)	500 m, 1.0 km, 1.5 km, 2.0 km and 2.5 km
Haemagogus leucocelaenus/ Haemagogus janthinomys	Percentage of native vegetation (2)	500 m, 1.0 km, 1.5 km, 2.0 km and 2.5 km
Haemagogus leucocelaenus/ Haemagogus janthinomys	Percentage of agricultural land (3)	500 m, 1.0 km, 1.5 km, 2.0 km and 2.5 km
Haemagogus leucocelaenus/ Haemagogus janthinomys	Number of forest fragments (4)	500 m, 1.0 km, 1.5 km, 2.0 km and 2.5 km
Haemagogus leucocelaenus/ Haemagogus janthinomys	Forest edge density (5)	500 m, 1.0 km, 1.5 km, 2.0 km and 2.5 km
Haemagogus leucocelaenus/ Haemagogus janthinomys	Number of native vegetation fragments (6)	500 m, 1.0 km, 1.5 km, 2.0 km and 2.5 km
Haemagogus leucocelaenus/ Haemagogus janthinomys	Native vegetation edge density (7)	500 m, 1.0 km, 1.5 km, 2.0 km and 2.5 km
Haemagogus leucocelaenus/ Haemagogus janthinomys	(4) + Cohesion index among forest fragments (8)	500 m, 1.0 km, 1.5 km, 2.0 km and 2.5 km
Haemagogus leucocelaenus/ Haemagogus janthinomys	(6) + Cohesion index among native vegetation fragments (9)	500 m, 1.0 km, 1.5 km, 2.0 km and 2.5 km
Haemagogus leucocelaenus/ Haemagogus janthinomys	(1) + (4)	500 m, 1.0 km, 1.5 km, 2.0 km and 2.5 km
Haemagogus leucocelaenus/ Haemagogus janthinomys	(5) + (3)	500 m, 1.0 km, 1.5 km, 2.0 km and 2.5 km
Haemagogus leucocelaenus/ Haemagogus janthinomys	(4) + (3)	500 m, 1.0 km, 1.5 km, 2.0 km and 2.5 km
Haemagogus leucocelaenus/ Haemagogus janthinomys	(4) + (8) + (3)	500 m, 1.0 km, 1.5 km, 2.0 km and 2.5 km
Haemagogus leucocelaenus/ Haemagogus janthinomys	(2) + (3)	500 m, 1.0 km, 1.5 km, 2.0 km and 2.5 km
Haemagogus leucocelaenus/ Haemagogus janthinomys	(2) + (7)	500 m, 1.0 km, 1.5 km, 2.0 km and 2.5 km
Haemagogus leucocelaenus/ Haemagogus janthinomys	(2) + (6)	500 m, 1.0 km, 1.5 km, 2.0 km and 2.5 km
Haemagogus leucocelaenus/ Haemagogus janthinomys	(7) + (3)	500 m, 1.0 km, 1.5 km, 2.0 km and 2.5 km
Haemagogus leucocelaenus/ Haemagogus janthinomys	(6) + (3)	500 m, 1.0 km, 1.5 km, 2.0 km and 2.5 km
Haemagogus leucocelaenus/ Haemagogus janthinomys	(6) + (9) + (3)	500 m, 1.0 km, 1.5 km, 2.0 km and 2.5 km
